# Performance Study of Chromium (VI) Removal in Presence of Phenol in a Continuous Packed Bed Reactor by *Escherichia coli* Isolated from East Calcutta Wetlands

**DOI:** 10.1155/2013/373412

**Published:** 2013-08-29

**Authors:** Bhaswati Chakraborty, Suvendu Indra, Ditipriya Hazra, Rupal Betai, Lalitagauri Ray, Srabanti Basu

**Affiliations:** ^1^Department of Biotechnology, Heritage Institute of Technology, Chowbaga Road, Anandapur, Kolkata 700107, India; ^2^Department of Food Technology and Biochemical Engineering, Jadavpur University, S. C. Mallik Road, Kolkata 700032, India

## Abstract

Organic pollutants, like phenol, along with heavy metals, like chromium, are present in various industrial effluents that pose serious health hazard to humans. The present study looked at removal of chromium (VI) in presence of phenol in a counter-current continuous packed bed reactor packed with *E. coli* cells immobilized on clay chips. The cells removed 85% of 500 mg/L of chromium (VI) from MS media containing glucose. Glucose was then replaced by 500 mg/L phenol. Temperature and pH of the MS media prior to addition of phenol were 30°C and 7, respectively. Hydraulic retention times of phenol- and chromium (VI)-containing synthetic media and air flow rates were varied to study the removal efficiency of the reactor system. Then temperature conditions of the reactor system were varied from 10°C to 50°C, the optimum being 30°C. The pH of the media was varied from pH 1 to pH 12, and the optimum pH was found to be 7. The maximum removal efficiency of 77.7% was achieved for synthetic media containing phenol and chromium (VI) in the continuous reactor system at optimized conditions, namely, hydraulic retention time at 4.44 hr, air flow rate at 2.5 lpm, temperature at 30°C, and pH at 7.

## 1. Introduction 

Environmental pollution due to structured and unstructured industrial growth and inadequate effluent treatment due to lack of awareness and insufficient treatment facility has become a serious health hazard in the world [1, 2]. Fresh water bodies are contaminated with different types of pollutants both organic and inorganic. One of the leading organic pollutants in water bodies is the phenolic compounds and the untreated metals, like chromium, which were another source of prolific water pollution. 

Chromium is a multivalent ion, among which Chromium (III) and Chromium (VI) form stable compounds. Chromium (VI) compounds (as in chromates, CrO_4_^−^, and dichromates, Cr_2_O_7_^−2^) [3] are mostly water soluble and are extremely toxic to human. It causes severe health hazards like allergic reactions, respiratory disorder, diarrhea, stomach and intestinal bleedings, cramps, and liver and kidney damage. Chromium (VI) is mutagenic in nature and leads to cancer [3–11]. Chromium (VI) is placed in the 16th position of the priority list prepared by the Agency for Toxic Substances and Diseases Registry (ATSDR) [12]. Thus Environmental Protection Agency (EPA), USA, has recommended the safety limit of chromium (VI) in potable water to be less than 50 *μ*g/L [2, 13], and for industrial discharge it is 5 mgL^−1^ [2]. The main source of chromium (VI) pollution of surface water is effluent from tanning, plastic, pigment, and paint manufacturing industries [5]. Industrial effluents like tannery effluent have been found to contain 80–250 mgL^−1^ chromium (VI) [2, 14]. Chromium (VI) can be removed both by chemical as well as biological processes. Chemical methods like precipitation, ion exchange, and electrochemical treatments have several disadvantages like incomplete conversion of chromium (VI) and ineffective removal of the metal from dilute metal solutions [15, 16] whereas biological detoxification of chromium (VI) can be done by cellular adsorption, conversion to nontoxic trivalent chromium, and bioaccumulation [15–20].

Several studies have been carried out for removal of chromium (VI) using microorganisms under laboratory condition [15–20]. In all cases, chromium (VI) had been used as the only stress factor in the growth media. Numerous tanneries are located in and around Kolkata whose effluents contain both chromium (VI) and phenol [21]. In the present study, attempt has been made to use microorganisms for removal of chromium (VI) in presence of an organic pollutant phenol which acted as the sole source of carbon. The bacterial strain used in the study was isolated from the East Calcutta Wetlands, the major waste degradation site for the metropolitan city Kolkata (previously known as Calcutta). The site received domestic waste of the city as well as industrial wastes, predominantly from the tanneries situated at the outskirts of the city. The performance of the reactor system under various hydraulic retention times, air flow rates, temperature, and pH conditions was studied, and reaction conditions were optimized.

## 2. Materials and Methods

### 2.1. Bacterial Strain Selection

A mixed bacterial consortium was isolated from soil of East Calcutta Wetlands, a Ramsar site in India. The mixed culture was then acclimatized in phenol using it as the sole source of carbon over a period of three months. 

Three pure strains were isolated from the mixed bacterial culture and identified by 16s rRNA method. Phylogenetic tree was constructed using Phylogenetic Tree Builder, and percentage homology was determined using ClustalW. Biochemical characterization was done following standard procedures. 

A comparative batch kinetic study of phenol degradation by individual bacterial species was carried out in presence of phenol as the sole source of carbon. The strain showing maximum phenol degradation rate was *Escherichia coli*. The strain, initially found to degrade phenol and remove chromium (VI) from medium separately, has been introduced to a continuous packed bed reactor. Media contained both phenol and chromium (VI). This study is engaged in checking the efficacy of this bacterial species for removal of chromium (VI) singularly as well as simultaneously with phenol to increase the efficiency of effluent treatment. 

### 2.2. Media Composition

The hexavalent chromium containing synthetic mineral salt (MS) media used in the experimental study is prepared from analytical grade chemicals procured from Merck, India. Either Phenol or glucose was used as source of carbon for growing the organisms. Composition of MS media (g/L) was potassium dihydrogen phosphate: 0.68; dipotassium hydrogen phosphate: 1.73; ferrous sulfate: 0.03; ammonium nitrite: 0.1; magnesium sulfate: 0.1; calcium chloride: 0.02; manganese sulfate: 0.03; glucose: 2.0. For using phenol as the source of carbon, 500 mg of phenol was added instead of 2 g glucose. Chromium (VI) was added to the media in form of potassium dichromate. 

### 2.3. Packed Bed Reactor System

The packed bed reactor was a bench scale reactor of volume 1000 mL. The clay chips used as packing material were derived from earthen tea cups used in West Bengal, a state in Eastern India. These tea cups were disposable and nonbiodegradable. They thus produced a huge bulk of solid waste. The bulk of solid waste could be reduced by recycling the clay chips. The used tea cups were collected from the vendors, washed, and dried under the sun. Then they were broken into smaller pieces. The broken pieces were sifted, and similar sized pieces were selected for packing the column reactor. The void volume (*ε*) was 0.81707, and the equivalent diameter (*D*_*p*_ = ^3^√(6*V*_*p*_/*π*), where *V*_*p*_ was mean volume of a chip in cubic centimeter) was 1.08 cm. The column reactor made of Borosil glass had an aspect ratio 10 : 1. The bed volume was 80% of the total reactor volume. 

The *E. coli* cells (24 h old) suspended in MS media containing 500 mgL^−1^ of phenol as the sole source of carbon were circulated through the reactor bed with the help of a peristaltic pump at the rate of 3 mL/min for 12 h through the inlet port located at 110 mm from the base of the reactor. 5 mL cell samples were collected before and after immobilization process, and their cell optical densities were measured at 600 nm by a UV-VIS spectrophotometer (Shimadzu). The difference of the two values was the cell loading factor. It was found that the *E. coli* cells were immobilized on the clay chips to 70% of its initial cell mass. The cells were allowed to grow on the clay chips for 5 days in the reactor by passing 500 mgL^−1^ of phenol through the reactor semicontinuously. The supernatant was drained out each morning for 5 days till sufficient cell growth occurs on the clay chips. 

First the chromium (VI) removal capability of the cells was studied using glucose as the sole source of carbon, such that only one toxic material, that is, chromium (VI), was present in the media. The purpose of the study was to check whether the organism can tolerate chromium (VI) in presence of a common source of carbon. The concentration of chromium (VI) in the MS media containing 2% glucose as the sole source of carbon was 500 mgL^−1^. The synthetic media was introduced into the reactor with the help of a peristaltic pump at different hydraulic retention times and air flow rates at 30°C and pH 7. The air was introduced into the reactor by a sparger in countercurrent direction, and the air flow rates were measured by a rotameter made of perspex. The system attained steady state when the input flow rate was equal to output flow rate of media. The two output sample collection ports were at 25 mm and 50 mm from the base of the reactor. At steady state the samples were collected from the lower port as well as the upper port, and the output concentrations of chromium (VI) were the same for both the ports for a single trial. For each hydraulic retention time and air flow rate, experimental trials were conducted in triplicate at a time interval of 15 mins. The hydraulic retention times were varied from 3.33 h to 8.88 h and air flow rates were varied from 1 lpm to 3 lpm. 

Glucose was replaced by phenol as the only source of carbon, and the cells thus had to biodegrade both phenol and chromium (VI). The synthetic media containing 500 mgL^−1^ of phenol and 500 mgL^−1^ chromium (VI) was introduced into the reactor with the help of a peristaltic pump. Air was sparged into the reactor in countercurrent direction to the substrate. The steady state system was studied at various substrate flow rates and air flow rates at room temperature simultaneously. The rate of removal of chromium (VI) from the synthetic media was measured spectrophometrically. Then further studies were done on the performance of the continuous reactor system in removal of Cr(VI) in presence of phenol. The reaction conditions were optimized based on removal efficiency of chromium (VI) at room temperature. Then the removal efficiency of the packed bed reactor was studied under the optimized substrate and air flow rates at different temperature and pH conditions.

### 2.4. Scanning Electron Microscopy (SEM) and Energy Dispersive X-Ray Spectrometry (EDS) Study

SEM and EDS were done with clay chips, bacteria immobilized clay chips, and bacteria immobilized on clay chips after treatment with chromium (VI) to obtain their topographical characterization and mineral composition. SEM photographs were taken after coating the samples with palladium by JFC-1600 Auto fine Coater with a scanning electron microscope (JSM 6360) using 17 kV. EDS was done to ascertain the presence of chromium using instrument INCA-MICS, 01736-03-04. 

### 2.5. Analytical Study

Phenol in the output sample was quantified spectrophotometrically at 490 nm using UV-VIS spectrophotometer (Shimadzu) by potassium ferricyanide and aminoantipyrine assay following the standard protocol (APHA) [22]. Chromium quantification was done spectrophotometrically at 540 nm using UV-VIS spectrophotometer (Shimadzu) with diphenylcarbazide following the standard procedure [22].

## 3. Results and Discussion

### 3.1. Selection of Strain from the Mixed Bacterial Consortium

The three bacterial strains constituting the mixed bacterial consortium identified by 16rRNA analysis were found to be *Psychrobacter sp.*, *Stenotrophomonas maltophilia*, and *Escherichia coli*.

**Figure 1:**
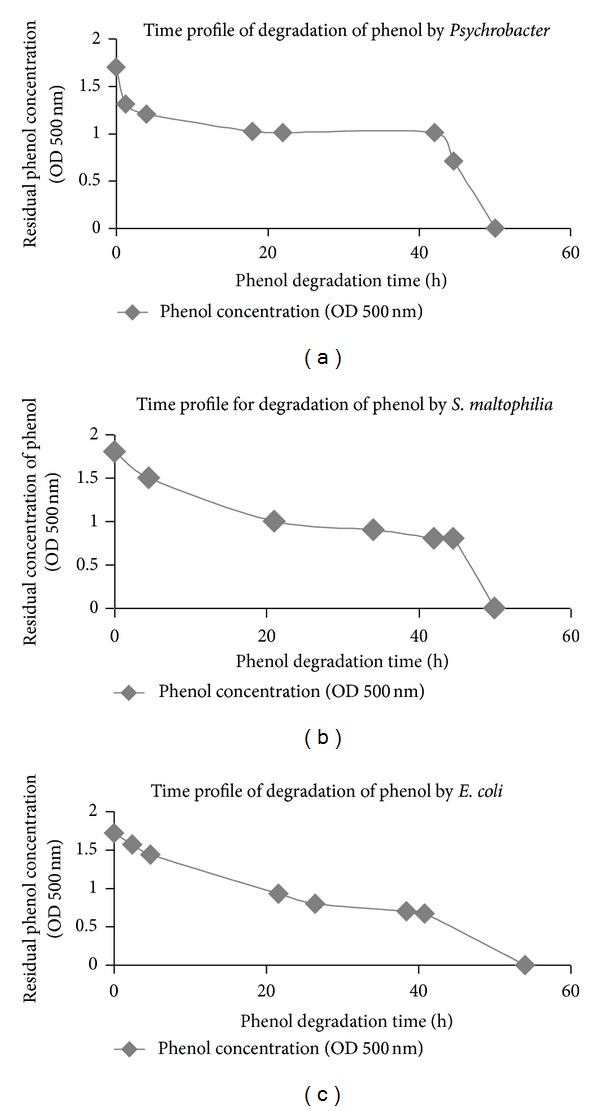
(a) Time profile of degradation of phenol by *Psychrobacter sp.* (b) Time profile of degradation of phenol by *S. maltophilia*. (c) Time profile of degradation of phenol by *E. coli*.

**Figure 2:**
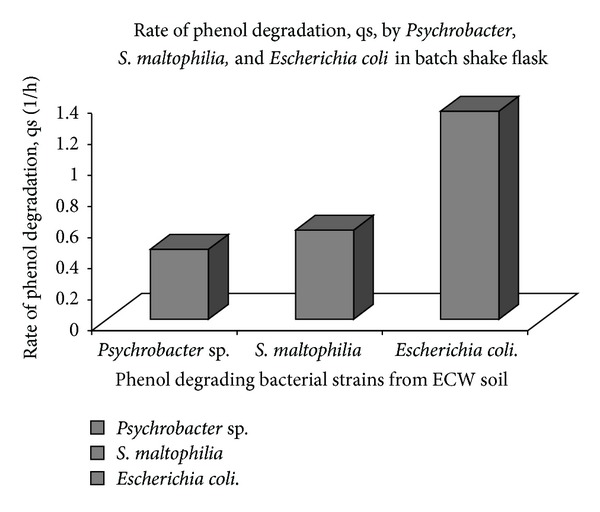
Comparative rate of phenol degradation by *Psychrobacter*, *Stenotrophomonas,* and *Escherichia coli*.

The rate of phenol degradation by each bacterial species was studied individually in batch process (from Figure 1) and compared as shown in Figure 2. *Escherichia coli* exhibited highest rate of phenol degradation (Figure 2) and was selected for further studies to measure its potential as a multiple toxic substance remover, in this case chromium (VI), in presence of phenol. 

**Figure 3:**
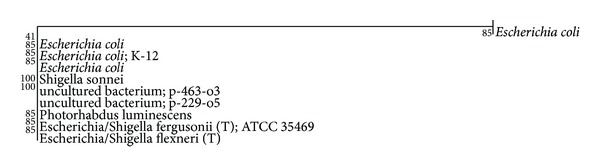
Phylogenetic tree of *E. coli* isolated from ECW soil with related genera of bacteria.

The 16s rRNA sequence of *E. coli* strain isolated from the soil sample of ECW was analyzed for strain identification, and the phylogenetic tree thus constructed to establish its homologous bacterial strains was shown in Figure 3. The microbe was found to be most similar to *Escherichia coli* (GenBank entry: AF233451). The next closest homologue was found to be *Escherichia coli*; K-12 (Genbank entry: M87049). In both cases the homology with the *E. coli* of present work isolated from ECW soil was found to be 100%. Results of the biochemical tests are given in Table 1. 

### 3.2. SEM and EDS Results

**Figure 4:**
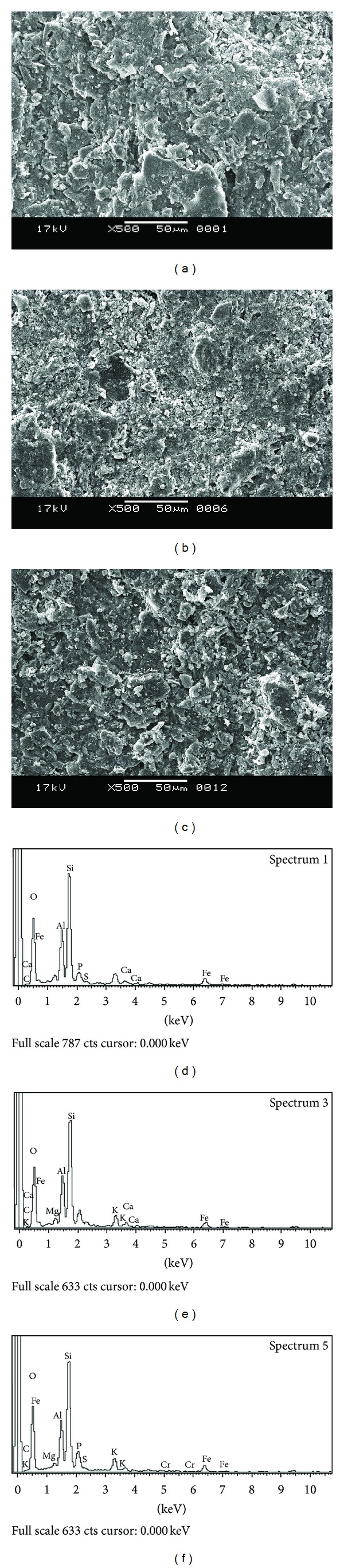
(a) SEM image of clay chip before immobilizing with *E. coli* cells. (b) SEM image of clay chip after immobilizing *E. coli* cells on to it. (c) SEM image of immobilized clay chip after treatment with phenol and chromium (VI). (d) EDS image of clay chip before immobilizing with *E. coli* cells. (e) EDS image of clay chip after immobilizing *E. coli* cells on to it. (f) EDS image of immobilized clay chip after treatment with phenol and chromium (VI).

SEM photographs of clay chips and clay chips containing immobilized *E. coli* cells are given in Figures 4(a) and 4(b). A polishing effect on the surface of clay chips was observed after immobilizing the *E. coli* cells.  Figure 4(c) represents the SEM photograph of clay chips containing *E. coli* cells after removal of chromium (VI). EDS profiles are given in Figures 4(d), 4(e), and 4(f). It was observed that carbon content increased with immobilization. Percentage of carbon content was 5.78 and 26.53% before and after immobilization, respectively. Chromium was absent in clay chips both with and without immobilized cells before treatment with chromium. Presence of chromium was reported (0.49% of the total elements) on the clay chips after treatment with chromium (VI). The result indicated that adsorption and bioaccumulation of chromium took place, and these mechanisms were responsible, at least partly, for removal of chromium. 

### 3.3. Removal Efficiency of Chromium (VI) Using Glucose as the Sole Source of Carbon

**Figure 5:**
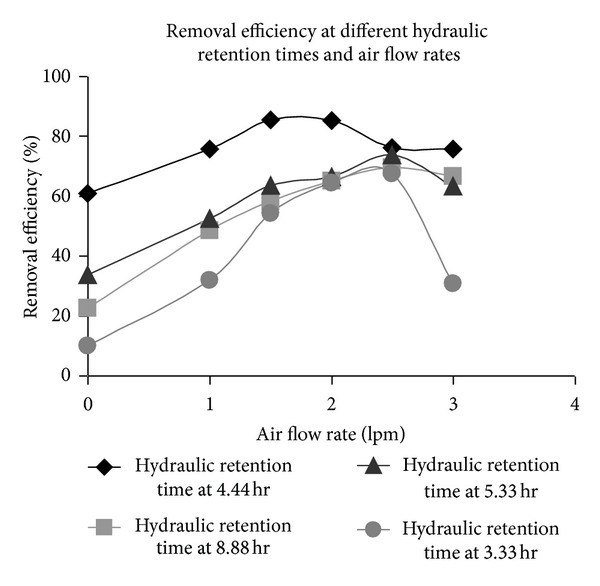
Removal efficiency of chromium (VI) by *E. coli* at different hydraulic retention times of glucose-containing medium and air flow rates.

MS media supplemented with 2% glucose as the sole source of carbon and 500 mgL^−1^ chromium (VI) was passed through the PBR immobilized with *E. coli* cells for determination of removal efficiency of chromium. The observations made on the runs conducted at different media-flow rates in a countercurrent packed bed reactor immobilized with *E. coli* were analyzed. The result showed increase in removal efficiency with increase in media flow rate till a threshold value. The removal efficiency of chromium (VI) was calculated for each substrate flow rate. It was observed from Figure 5 that the removal efficiency increased as hydraulic retention time (HRT = *V*/*Q*, where *V* = effective volume of reactor in mL and *Q* = substrate flow rate in mL/min) increased for relatively lower HRT of substrate, but at higher HRT values the removal efficiency decreased. This could be because of an existence of an external diffusion layer and also saturation of cellular metabolism. The rate of transfer of substrate through the diffusion was inversely proportional to the thickness of this layer. On the other hand, the thickness of this layer was inversely proportional to the substrate flow rate through the reactor [23, 24]. The hydraulic retention time of the substrate in the reactor was inversely proportional to substrate flow rate. Thus the rate of diffusion of substrate to the *E. coli* cells decreased at higher hydraulic retention time [24]. As a result the removal efficiency of chromium (VI) effectively decreased at higher HRT. The maximum removal efficiency was 85% with hydraulic retention time of 4.44 h (Figure 5). 

### 3.4. Effect of Air Flow Rates in Presence of Glucose on Chromium (VI) Removal Efficiency

The effect of rate of aeration on removal efficiency of chromium (VI) was studied as *E. coli* is an aerobic organism. For aerobes, the rate of metabolic activities depends on rate of oxygenation. Thus the air flow rate was varied from 0 lpm to 3 lpm for each glucose substrate flow rate. The rate of Chromium removal increased with air flow rates till 2–2.5 lpm. But beyond that the rate of removal of chromium (VI) decreased due to profuse foaming. Foaming caused changes in both the size and composition of the air bubble. It altered the dissolved oxygen profile due to heterogeneous dispersion in the reactor volume. Due to increased residence time of the air bubble in the reactor the air bubble became oxygen depleted; CO_2_ accumulated in the air bubble replacing the oxygen. CO_2_ is toxic to cells and inhibits their metabolic activities. Thus accumulation of CO_2_ in the reactor space due to foaming decreased the removal efficiency of chromium (VI) by *E. coli* cells. The maximum removal efficiency (Figure 5) was at the hydraulic retention time of glucose 4.44 h with an air flow rate of 2 lpm. The maximum chromium (VI) removal efficiency was found to be 85% in glucose medium.

### 3.5. Removal Efficiency of Chromium (VI) Using Phenol as the Sole Source of Carbon

**Figure 6:**
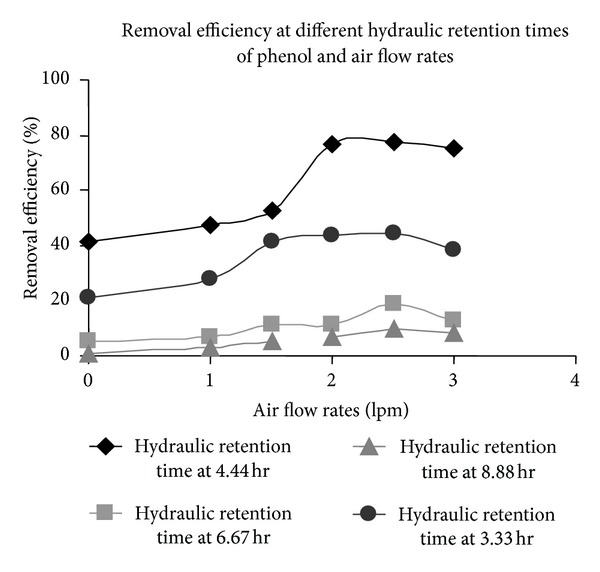
Removal efficiency of chromium (VI) by *E. coli* at different hydraulic retention times of phenol and air flow rates.

The synthetic media used for the study was prepared by dissolving phenol (500 mgL^−1^) and chromium (VI) (500 mgL^−1^) in MS media. Phenol was the sole source of carbon present in the media for the immobilized *E. coli* cells. The substrate flow rate was varied from 1.5 mL/min to 4 mL/min; that is, the hydraulic retention time (HRT = *V*/*Q*, where *V* = effective volume of reactor in mL and *Q* = substrate flow rate in mL/min) was varied from 3.33 h to 8.88 h. The effect of phenol flow rate on chromium (VI) removal efficiency was similar to the effect of glucose flow rate. The best removal of chromium (VI) occurred at HRT value of 4.44 h as was observed from Figure 6.

### 3.6. Effect of Air Flow Rates in Presence of Phenol on Chromium (VI) Removal Efficiency

The *E. coli* cells are aerobic in nature; thus aeration of the reactor is a primary necessity to sustain the culture. To note the effect of rate of aeration on the removal efficiency of chromium (VI), the air flow rates were varied as 0 lpm, 1 lpm, 1.5 lpm, 2 lpm, and 2.5 lpm for each substrate flow rate. The removal efficiency profile with respect to air flow rates was studied as shown in Figure 6, and it was observed that removal of chromium (VI) increased with increasing air flow rates till the process became independent of rate of oxygenation. With further increase in aeration rate it was found that removal efficiency reduced due to excessive foaming in the reactor leading to insufficient gas-liquid interfacial diffusion [25].

The maximum chromium (VI) removal efficiency of 77.7% was observed at a phenol-chromium (VI) hydraulic retention time of 4.44 hr with an air flow rate of 2.5 lpm in the countercurrent continuous packed bed reactor. Requirement of higher air flow rate for removal of chromium in presence of phenol could be explained in the following manner. Glucose is the most easily metabolized source of carbon. On the other hand, phenol has inhibitory action on cells. Moreover, conversion of phenol to TCA cycle intermediates requires action of oxidase which utilizes molecular oxygen. Thus metabolism of phenol consumes more oxygen than glucose. As a result, higher flow rate of oxygen is required to have the same chromium removal in presence of phenol than glucose. 

### 3.7. Effect of Temperature on the Performance of the Continuous Packed Bed Reactor

**Figure 7:**
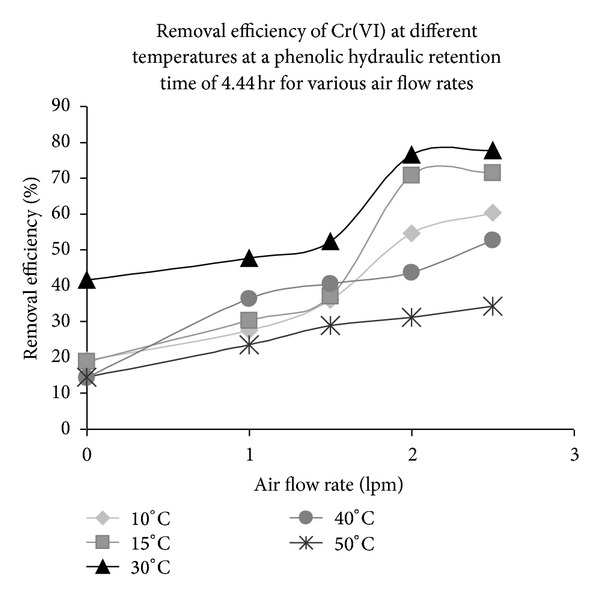
Removal efficiency of chromium (VI) by *E. coli* at different temperatures and air flow rates at phenolic chromium (VI) media and HRT of 4.44 h.

The performance efficiency of the reactor system containing both phenol and chromium (VI) as pollutants was studied at different temperatures from 10°C to 50°C. The immobilized cells were fed with MS media containing 500 mgL^−1^ chromium (VI) and 500 mgL^−1^ phenol at a hydraulic retention time of 4.44 h. The air flow rates were varied from 0 lpm to 2.5 lpm. The temperature of the media was maintained at 10°C, 15°C, 30°C, 40°C, and 50°C, respectively and removal efficiency of chromium (VI) was measured for each temperature. The system proved to be in working condition and was able to remove chromium (VI) at both the limiting temperature conditions. *E. coli* is a mesophilic organism, but there are reports on survival/adaptation of *E. coli* in extreme temperatures [26–28]. It has been reported that protein profile of an *E. coli* cell changes with change in temperature (tested between 13.5°C and 46°C) which makes the cell adapt to different temperatures [28]. The rate of removal was significantly less as compared to the rate of removal at 30°C under similar air flow rates as evident from Figure 7. The rate of degradation of *E. coli* proteins increased at temperatures above 37°C [28], and it was observed that the rate of removal of chromium (VI) was lowest at 50°C for all air flow rates.

### 3.8. Effect of pH on the Performance of the Continuous Packed Bed Reactor

**Figure 8:**
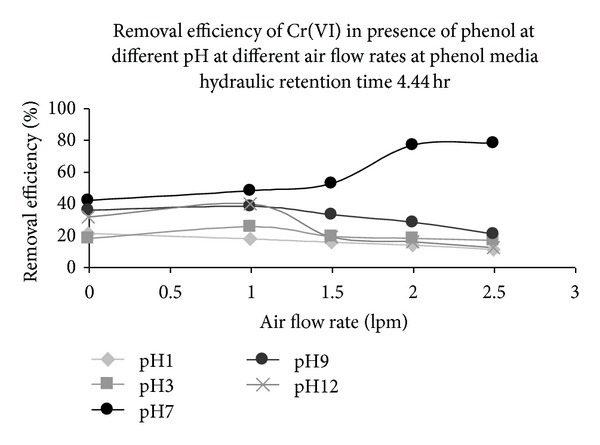
Removal efficiency of chromium (VI) by *E. coli* at different pH and air flow rates at phenolic chromium (VI) media and hydraulic retention time of 4.44 h.

For the hydraulic retention time of phenol and chromium(VI) at 4.4 h, pH of the synthetic media containing phenol and chromium (VI) was varied from pH 1 to pH 12. The effect of pH was studied under different aeration rates from 1 lpm to 2.5 lpm, as shown in Figure 8. *E. coli* was seen to remove chromium even at pH 1 and pH 3. Maximum chromium (VI) removal efficiency of 77.7% was observed at 2.5 lpm air flow rate at pH 7. Reports corroborative of our observation of survival of *E. coli* at extreme pH conditions were available [29–31]. 

The optimum pH for growth of *E. coli* is pH 7.0. The strain could survive at extreme pH conditions under lower air flow rate. At higher air flow rate, foaming started, and removal of chromium (VI) was reduced even with the minimum foam formation under extreme pH conditions. This may be due to the fact that the growth of organisms was reduced under the dual stress caused by extreme pH as well as carbon dioxide accumulation in the system due to foam formation. 

Previous studies conducted by other researchers on removal of chromium (VI) by different bacterial species have shown removal efficiencies to vary from 24.2% to 74.9% [32–35], in batch culture and without presence of a second stress element in media. Previous works showed 48% removal of 500 mgL^−1^ chromium (VI) over a period of four days and 40% removal in biofilter mode for the same initial chromium (VI) concentration in batch culture [32]. Some other researchers removed up to 74.9% of the influent chromium concentration of 100 mgL^−1^ [34]. Rengifo-Gallego et al. attained a removal efficiency of 62.85% using bacterial consortium for an initial chromium (VI) concentration of 5 mgL^−1^ in a bioreactor [35]. Most of the work done on removal of chromium was done using glucose as the sole source of carbon. On the other hand, the present study reported chromium removal of 85% in presence of glucose in the media, which is much higher than the other studies reported and 77.7% in presence of phenol, the second stress factor, in the media in a continuous countercurrent packed bed reactor. 

## 4. Conclusion

The *E. coli* strain isolated from a mixed bacterial consortium from soil of East Calcutta Wetlands could remove chromium utilizing phenol as the sole source of carbon continuously in a packed bed reactor using clay chips as the immobilizing matrix. The optimum conditions for operating the reactor were HRT: 4.44 h; air flow rate: 2.5 lpm; temperature: 30°C, and pH: 7. The maximum removal efficiency of chromium (VI) under the above optimized condition was 77.7%. The system has potential for treatment of mixed waste containing both organic pollutants and heavy metals present in tannery effluent.

## Figures and Tables

**Table 1:** Biochemical characterization of *E. coli*.

Biochemical tests	Response of *E. coli* isolated from ECW soil
Gram staining	−
Lactose	+
Mannitol	+
Methyl Red	+
Voges-Proskauer	−
Citrate	−
Gelatin	−
Acid-fast	+
Oxidase	−
Catalase	+
